# Development and Validation of the Psychotherapeutic Effectiveness Attribution Questionnaire (PEAQ-12) in a Spanish Population

**DOI:** 10.3390/ijerph181910372

**Published:** 2021-10-01

**Authors:** Antonio Romero-Moreno, Alberto Paramio, Serafín J. Cruces-Montes, Antonio Zayas, Diego Gómez-Carmona, Ana Merchán-Clavellino

**Affiliations:** 1 Department of Psychology, Faculty of Education Sciences, University of Cádiz, 11519 Puerto Real, Spain; antoniofrancisco.romero@uca.es (A.R.-M.); serafin.cruces@uca.es (S.J.C.-M.); ana.merchan@uca.es (A.M.-C.); 2 University Institute of Research in Social Sustainable Development, University of Cadiz, 11405 Jerez de la Frontera, Spain; diego.gomezcarmona@uca.es; 3 Department of Marketing and Communication, Faculty of Social Sciences, University of Cadiz, 11405 Jerez de la Frontera, Spain

**Keywords:** attribution, psychotherapist, scale, validation, development

## Abstract

In recent decades, the study of psychotherapy effectiveness has been one of the pillars of clinical research because of its implication for therapeutic cure. However, although many studies have focused their interest on the patient’s perception, there are no instruments oriented to the study of psychotherapists’ attributions of effectiveness: to what factors psychotherapists attribute responsibility for the cure of the therapies they provide. The present study aimed to develop and validate an instrument for assessing the attribution of the effectiveness of psychotherapy in a population of 69 psychotherapists of different theoretical orientations. After an initial process of inter-judge content validation, 12 items were selected for validation in the targeted population, adequately fulfilling the quality requirements in the validity–reliability tests, and grouped into four factors after principal component analysis. These factors were as follows: (1) therapeutic alliance enhancers; (2) psychotherapist emotional characteristics; (3) therapy-specific variables; and (4) facilitators of patient engagement with therapy. This four-factor structure also showed a good fit for the fit indices checked in confirmatory factor analysis. In summary, we can conclude that the Psychotherapeutic Effectiveness Attribution Questionnaire (PEAQ-12) developed in our research can be helpful if tested on a larger number of individuals. The results can be replicated in other populations of psychotherapists.

## 1. Introduction

The study of the variables involved in the effectiveness of psychotherapy has occupied ample space in psychotherapeutic research in recent decades. The possible elements that may affect psychotherapy outcomes have usually been classified into two broad categories: psychotherapy-specific variables and common factors (or non-specific variables) [1,2,3,4]. The specific variables refer to genuine and idiosyncratic behaviours in each therapy modality, making them recognisable and different from each other. We refer to the particular techniques and procedures used and the therapeutic approach employed based on a specific theoretical orientation. Common variables point to active ingredients present in all psychotherapies and are instrumental in therapeutic change [5]. Such variables common to different psychotherapies would include those related to the patient, with the effect of the therapist on the therapy and the therapeutic interaction [6,7].

Traditionally, specific variables were attributed full responsibility for the healing process [8]. However, Lambert et al. [9] set the contribution of specific therapeutic techniques at only 15%, a figure with which other authors concur [10,11,12]. Wampold’s [13] meta-analysis assigned therapist-performed techniques 13% of the change variance, which Duncan [14] set at only 1%. Cuijpers et al. [15] established that specific factors were only responsible for 17% of patient improvement in their meta-analysis.

In recent research on therapeutic outcomes and processes, there seems to be a broad consensus that common factors are primarily responsible for therapeutic change [11,16,17,18,19,20,21,22]. Thus, research on common factors indicates that 85% of change is due to such elements present in all psychotherapy modalities [23,24]. However, it is important to note that the common factors are not just a set of therapeutic elements present in all or most psychological treatments, but rather a theoretical model of the mechanisms of psychotherapeutic change [25].

Of all the common variables, those related to the therapeutic relationship or interaction (and, specifically, the therapeutic alliance that occurs within it) are among those that contribute most to the effectiveness of psychological treatments [26,27,28,29].

In this sense, studies such as Gaston et al. [30] or Luborsky et al. [31] found that the therapeutic alliance explained between 36% and 57% of the variance in the therapy outcome. Safran and Segal [32] concluded that 45% of therapeutic change was due to factors related to the therapeutic relationship. A review of 132 research studies by Orlinsky et al. [29] concluded a strong relationship between the quality of the therapeutic alliance and psychotherapy outcome. Thus, a large body of research emerging worldwide establishes that various aspects of the therapeutic alliance correlate positively with treatment outcomes [33,34]. Thus, developing a close patient–therapist bond is considered a crucial component of successful psychotherapy [35,36,37,38,39,40,41].

Patient-related variables are also considered extremely important [42], as there are studies that estimate their contribution to therapeutic success at up to 40% of the variance [9]. However, demographic factors (age, socioeconomic status, educational level, race or gender) or those related to the locus of control do not play a significant role in psychotherapy outcomes, or contradictory data are obtained in different studies [43,44,45]. Crucial elements in the therapeutic healing process are the patient’s expectation of cure, the patient’s involvement in the course of therapy and the faith and credibility the patient assigns to the therapist.

The study of patient expectations, even from the first days of therapy, has been one of the best and longest studied variables, being considered a crucial element in the healing process [11,46,47,48]. On the other hand, treatment involvement is a powerful indicator of the therapeutic alliance in the treatment process [49]. As the patient is an active agent of change [50], treatment adherence is an essential element in explaining the success of an intervention [51,52]. The patient’s expectation of cure and involvement in treatment could be assimilated to what other studies have called motivation to change or motivation to follow treatment [53]. Finally, the faith and credibility the patient gives to the therapist are shown to be fundamental factors in the expected results [54], both for maintaining a positive belief towards the treatment and the techniques used and for the trust established with the psychotherapist.

Regarding common therapist variables, that is, variables related to the effect of the therapist, a large number of them are relevant. Regarding the therapist’s emotional well-being, the therapist’s level of emotional adjustment is related to therapeutic success [55], further noting that a disturbed therapist may impede their patients’ growth and induce negative changes in them [28,56,57]. The empathy shown has also been related to better adherence to the patient’s treatment [58,59,60,61]. The absence of empathy and the therapist’s lack of understanding of the problem were conducive to the patient’s therapeutic desertion [62,63]. Regarding the degree of acceptance, interest, understanding and encouragement shown by the therapist to the patient, it has been established that the psychotherapist must have an attitude that favours a therapeutic climate that facilitates change based on listening to, understanding and accepting the patient [1,51,64]. However, it is just as important to show a willingness to listen and understand the patient as it is to make patients feel listened to and understood [65].

Another component to consider is the therapist’s directivity/support, understood as the degree to which instructions, information and specific help are provided and tasks are structured and delimited [66]. To obtain favourable results, a good therapist must modulate their directivity and support depending on the phase of treatment, the type of problem addressed in the consultation and the patient’s personality characteristics [66,67,68,69].

Similarly, the therapist’s perception of patient involvement is mentioned as one of the main desirable characteristics in therapists [70,71,72,73]. According to the study by Lafferty et al. [60], more effective therapists felt that their patients were less involved in their treatment and made less progress, whereas less effective therapists perceived greater involvement and improvement.

The variable of the therapist’s ability to influence the patient, which is also present in other health professionals, seems to influence the outcome and effectiveness of treatment [74,75,76], where the therapist’s ability to persuade is established as a crucial element [77].

The last therapist variable to consider is the therapist’s experience. In general, the therapist’s expertise is related to positive changes [57]. Thus, older and more experienced therapists tend to act with more empathy and tolerance than beginners towards patients’ expressions of negative emotions related to the development and improvement of the therapeutic alliance [75,78].

Thus, the scientific evidence shows that it is the common elements present in all psychotherapy, regardless of the theoretical model on which it is based, that explains its effectiveness to a greater extent, meaning both of the variable types should be taken into consideration in terms of theory and research and practice [25]. Based on this evidence, it is interesting to question to what extent therapists have assumed the fundamental role that common factors play in the healing process, or whether, on the contrary, as Botella and Feixas [79] pointed out, it is to be expected that they will continue to point to specific variables as the main precursors of therapeutic change.

This separation between research and praxis (already pointed out by Beitman, 1987 [80]) can have repercussions on therapeutic success in such a way that the therapist targets strategies that are not as effective as those recognised by scientific evidence. For example, it is interesting that the therapist takes responsibility for their mental health and self-care, making it easier to respond therapeutically to patients’ problems [26]. Furthermore, flexibility is considered a fundamental quality of the therapist [42] and their ability to persuade. Although psychological treatments are usually highly protocolised, their effectiveness depends on a high degree of these skills [77]. The therapist’s willingness to listen to and understand themselves is also crucial [81], as the reactions and associations to the material brought by the patient are crucial information for understanding the dynamics of the client [82].

For all these reasons, and with the intention to develop programmes for the training of psychotherapists and for helping them to establish strategies that improve the success of their treatments, it is necessary to draw up a questionnaire to detect the variables to which they attribute the most significant responsibility in the process of therapeutic change. The aim is for therapists to be aware of any incorrect notions or ingrained attitudes they may have in order to correct them, and to favour the establishment of the focus of their intervention on the axis of the variables of the patient, the therapist and the therapeutic interaction, considered by psychotherapeutic research to be the main providers of effectiveness. Ultimately, the construction of this assessment tool would help therapists to check whether the therapeutic elements to which they attribute the greatest effectiveness are those that, in fact, bring the greatest efficacy to psychotherapy, which would ultimately help them to assign an appropriate weight to the different active components present in the treatments they carry out.

## 2. Methods

### 2.1. Sample

The study population chosen was all psychotherapists included in the directory of the Official College of Psychologists of Western Andalusia (Spain). A total of 69 psychotherapists (50.7% male and 49.3% female; mean age = 41.5, SD = 6.41) participated in the validation of the instrument. The theoretical orientation of these psychotherapists was divided into 4 orientations: 44.9% cognitive-behavioural, 26.1% psychodynamic, 15.9% eclectic, 10.1% humanistic-systemic, and those who chose not to declare their theorical orientation 3%.

### 2.2. Instrument

A questionnaire was sent to a selected group of 12 experts in psychotherapies and psychological treatments, who were asked to collaborate to assess the degree of congruence in the assignment of the different items to the objectives proposed. The composition of the questionnaire comprised a wide variety of items, coded and closed-ended (Annex 1). As a preliminary step in developing the questionnaire, a thorough literature review on outcome research and therapeutic processes was carried out to include the relevant variables in the form of items. Once those variables that have been analysed with the greatest emphasis as possibly being responsible for cure had been selected, the items corresponding to them were drafted, and their content was validated using the procedure described by Osterlind [83] by means of expert judgement.

The items of the selection before expert judgement corresponded to each of the psychotherapeutic variables relevant to the healing process and to which the psychotherapists had to assign a rating from 1 to 5 (where 1 = “does not influence at all on the patient’s improvement” and 5 = “has a great influence”). These variables were as follows: (1) therapeutic approach used; (2) techniques or procedures used; (3) patient’s expectation of cure; (4) patient’s involvement in the therapy; (5) patient’s faith and credibility assigned to the therapist; (6) therapist’s emotional well-being; (7) empathy shown by the therapist; (8) directivity and support shown by the therapist; (9) therapist’s perception of the patient’s involvement; (10) therapist’s ability to influence the patient; (11) degree of acceptance, interest, understanding and encouragement shown by the therapist to the patient; (12) experience of the therapist; (13) establishment of a therapeutic alliance between the therapist and patient. In addition to the scale, items related to the following variables were incorporated in order to describe the study population:

- Demographic characteristics: sex and age, level of studies and place of practice of psychotherapy;
- Therapist clinical characteristics: experience as a psychotherapist, theoretical orientation and access to publications on psychotherapy research.

### 2.3. Data Collection

The sample selected for the study was sent a letter of introduction, informed consent and the final version of the scale (Appendix A). It was facilitated by email because the participants resided in distant locations. Respondents were informed that feedback would be provided to them after presenting the study in response to their collaboration.

### 2.4. Ethical Considerations

This study was conducted in compliance with the Declaration of Helsinki of 1975. An information letter was sent to all participants together with the questionnaire. The participants were guaranteed confidentiality and anonymity. All participants signed the informed consent.

### 2.5. Data Analysis

The normality of the quantitative variables was verified using the Kolmogorov–Smirnov–Lilliefors test [84]. Descriptive statistics were used to summarise demographic data. Means and standard deviations were examined to determine items and overall score distributions.

For content validity, the congruence index of each item with the objective it intends to measure was calculated [85,86], selecting those that achieved a higher score to form part of the instrument (I_ik_ > 0.5).

Construct validity was assessed using exploratory factor analysis (EFA) and the known group technique [87]. The EFA was undertaken [88] using principal component analysis (PCA). Varimax rotation was selected as this minimises the number of factors needed to explain each variable to obtain a clearer factorial structure. The rotated factor matrix was examined to identify the items that load on factors. Items with factor loadings > 0.40 have great practical relevance and define the factors properly [89] and were thus extracted. Previously, factorability was assessed through the Kaiser–Meyer–Olkin (KMO) test and Bartlett’s test of sphericity [90]. Based on the known groups technique, the responses were compared between the different psychotherapeutic orientations. Construct validity was also assessed with confirmatory factor analysis (CFA) to verify the factor structure of our sets of observed variables. Based on the recommendations by Bentler and Chou [91], who suggest at least five subjects for each free parameter, 69 subjects were considered adequate to test the single- and four-factor models of the scale with CFA. A maximum likelihood estimator of model parameters and a full maximum likelihood approach (FIML) [92] were used to manage missing data. We used several fit indices to assess the model fit (Table 1), as suggested by Kline [93].

Contrasting group validity was tested by comparing self-reported wine relationship items among participants with one-way ANOVA.

Internal consistency was assessed by determining the Cronbach’s alpha coefficient, corrected item-to-total score correlations using Pearson correlation coefficients and alpha estimation when an item was removed from the scale [98]. The criterion used for acceptable overall internal consistency was a value between 0.70 and 0.90 of Cronbach’s alpha, which is considered adequate for instruments used in research, whereas values above 0.90 may suggest redundancies in the scale [99]. The proportion of respondents with the lowest (1 point) or highest possible score (5 points) was calculated to examine the presence of floor and ceiling effects. Values higher than 20% were considered a moderate effect and higher than 50% as major [100].

Internal consistency reliability of the scale was estimated considering the following indices:

1. Greatest Lower Bound (glb) [101].
2. McDonald’s omega (ωt) [102];
3. Standardised Cronbach’s alpha (α) [103].

The results were considered statistically significant if the *p*-values were <0.05. Statistical analyses were performed using SPSS Statistics version 25, except for the CFA, which was performed with AMOS version 25.

## 3. Results

### 3.1. Content Validity

The three items considered by the judges as the most congruent with the proposed objectives were as follows: “therapist experience” with the complete agreement of the judges (I_ik_ = 1), “patient’s expectation of cure” and “patient involvement” (both with I_ik_ = 0.916). The variable “emotional well-being of the therapist” was eliminated as it did not reach the minimum level required for acceptance in the questionnaire (I_ik_ = 0.16).

The remaining variables reached a level above the minimum level (I_ik_ > 0.5), meaning that a total of 12 items were finally selected for inclusion in the questionnaire (Table 2).

### 3.2. The Overall Scale and Item Score Distribution

The mean overall score of the scale in the sample was 3.96 (SD = 0.46). All items displayed a short range of median values (3.0–5.0). The mean for each item is presented in Table 3, together with the floor and ceiling effects. Eight items presented a major ceiling effect, but their presence was justified due to the variation in the internal reliability if the items were removed.

### 3.3. Internal Consistency

The Cronbach’s alpha coefficient was 0.727 for the whole scale. This value is accepted due to the number of items [104,105]. Table 1 shows Cronbach’s alpha when an item was deleted.

The factors finally presented the following reliability indices: 0.669 on the first factor; 0.621 on the second factor; 0.757 on the third factor; and 0.348 on the fourth factor. No items were eliminated in the four subscales as no significant gains in reliability were obtained, and even the elimination of items with marginal loadings decreased the reliability. Thus, the estimated reliability for the subscales corresponding to the four factors was, taking into account the number of items, optimal for three of them, obtaining fairly acceptable alpha values for the first three factors. For the fourth factor, an alpha value of 0.348 was obtained, which is considered low. However, due to the small number of items, Cronbach’s alpha values of this magnitude are acceptable [104].

This can be proved if we equate the four subscales to a larger number of items (e.g., to 12, as in the full scale). Following the Spearman–Brown formula, in this case, we would obtain the following reliability indices for the four subscales: 0.83 for the first factor; 0.86 for the second; 0.95 for the third; and 0.76 for the fourth.

### 3.4. Inter-Rater Reliability

The ICC for the overall scores reached a value of 0.727 (95% confidence interval = 0.616–0.817; *p* = 0.000) for no interaction effect, and for each item, it ranged from 0.118 to 0.271. The results obtained indicate that the responses were very stable for both the full scale and all items.

### 3.5. Known Groups Technique

The difference between orientations was highly statistically significant (F = 2.44; *p* = 0.035). In addition, the cognitive-conduct orientation reached higher mean scores in every single item (except for item 11: therapist’s experience), and this difference was significant for all items (*p* < 0.05), except for items 3, 6, 8 and 10 (techniques or procedures used, therapist’s empathy, therapist’s perception of patient involvement and therapist’s degree of acceptance, interest, understanding and encouragement, respectively).

### 3.6. Exploratory Factor Analysis

**Figure 1:**
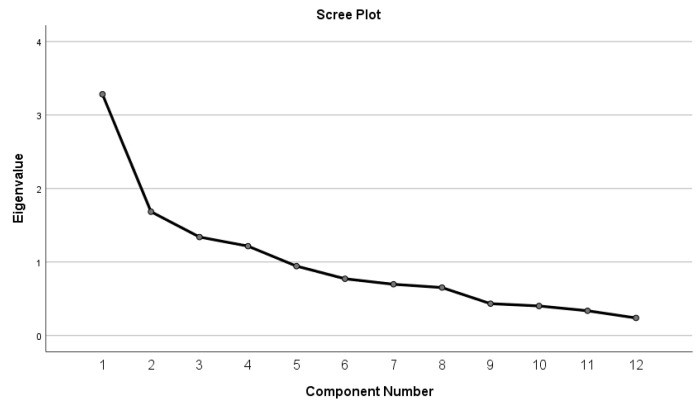
Sedimentation graph of factor components.

The significance of Bartlett’s test of sphericity (X^2^ = 181.345; df = 66; *p* = 0.000) and the size of the KMO measure of sampling adequacy (KMO = 0.72) revealed a common variance of the items of the scale suitable for factor analysis [106]. The PCA and varimax rotation revealed four factors with eigenvalues exceeding 1 (Figure 1). The first component had an eigenvalue of 3.282, which explained 27.351% of the variance, the second one had an eigenvalue of 1.685, explaining 14.039% of the variance, the third one had an eigenvalue of 1.34, explaining an additional 11.166% of the variance, and the fourth one had an eigenvalue of 1.216, explaining 10.136% of the variance. The rotated factor matrix was examined to identify items that loaded on these factors (Table 4).

### 3.7. Confirmatory Factor Analysis

**Figure 2:**
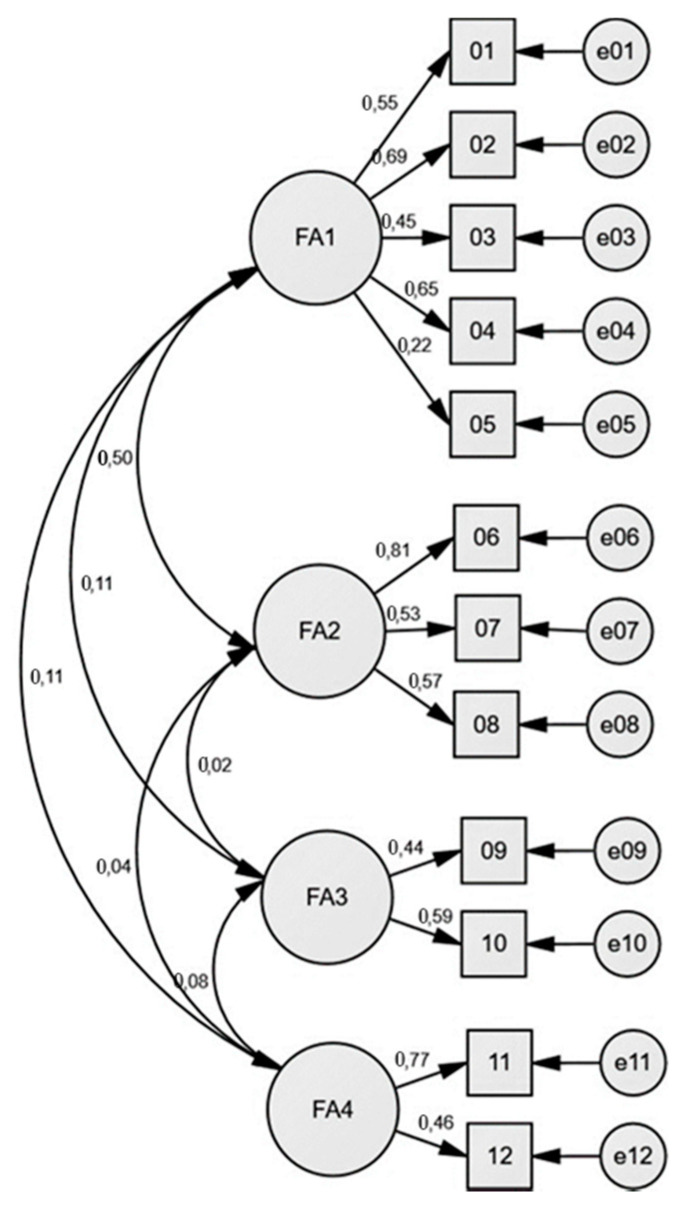
The four-factor model diagram with standardised estimates.

The four-factor model (Figure 2) showed the following fit indices: χ^2^(84) = 48.47, *p* = 0.000; CFI = 0.995; TLI = 0.992; RMSEA = 0.012 (90% CI = 0.000–0.080); SRMR = 0.043. The correlations among the four factors also indicated a high percentage of variance shared by the factors. The loadings of the selected model are presented in Table 3.

## 4. Discussion

The present study developed and validated a questionnaire that allows for detecting the most responsible variables for the process of therapeutic change. The idea is to try to help therapists to establish strategies to improve the success of their treatments. To this end, the aim is to provide an instrument for therapists, through its administration, to find out their attributional biases in terms of the importance they attach to the specific factors of the therapies they practice (techniques and procedures used) and to the common factors of the therapeutic process (variables involving the patient, the therapist and the interaction between them). Once the attributed errors have been detected, the therapist can focus on enhancing the common skills and attitudes that, according to psychotherapy research, are responsible for greater effectiveness in a psychotherapeutic intervention [11,19,20,22].

Firstly, it can be confirmed that there is an adequate level of content validity of the core variables that make up the questionnaire, corresponding to 12 items (I_ik_ > 0.5), according to [83] the congruence index. The 12 judges, experts in psychotherapeutic processes and clinical or health psychology, assessed the congruence between each indicator and the domain it measures. After a thorough review of the literature, 13 items were initially selected. All items obtained adequate levels of congruence, except for “emotional well-being of the therapist”, which was eliminated. It did not reach the minimum level required for acceptance in the final questionnaire (I_ik_ = 0.16).

Similarly, the questionnaire presents adequate levels of reliability, showing a Cronbach alpha of 0.727 for the total scale, meeting the criterion of being between the range of 0.7 and 0.9 [99]. Moreover, it should be taken into account that it is a scale with a small number of items, meaning that such an internal consistency is considered more than satisfactory for that number of items [105] and would correspond, following the Spearman–Brown attenuation formula, to an alpha value of ≈ 0.9, for an instrument with a number of items equal to 36.

However, in the analysis of the discriminatory capacity of each item, it was observed that several do not meet the criteria established between the total score and the item by means of the Pearson correlation coefficients, identifying ceiling percentages of over 20% in a total of 10 items, with 3 items of these with scores of over 50% and thus showing a more significant effect. If we eliminate some of the items, the reliability of the total scale is lost.

Concerning Cronbach’s alpha and the criticism that it is the best parameter to determine internal consistency [107], two more scores were calculated: the greatest lower bound (GLB) coefficient [101] and the coefficient ω [102]. In general, they scored very close to 1, which is an adequate level of reliability, with a difference between these coefficients of only 0.10. Despite this, and according to the estimates established on when it is appropriate to use one or the other coefficient [108], we assume values of ω = 0.79 and α = 0.71, as these two coefficients are more appropriate when the total test scores are normally distributed, with ω even being the first option, followed by α, as they avoid the problems of overestimation presented by the GLB coefficient. The last coefficient is recommended when the proportion of asymmetric items is high, whatever the sample size.

As for construct validity, first, all tests of suitability for factor analysis, the KMO (0.72), Bartlett’s test of sphericity (*p* < 0.05) and the determinant matrix (0.042), were met. The exploratory analysis confirmed four factors that explain 62.7% of the variance with adequate levels of reliability. Therefore, our research shows that the questionnaire, elaborated to elucidate the attributions of effectiveness in the psychotherapeutic process, can be divided into four dimensions, related to the specific variables of psychotherapy and the common factors (or non-specific variables) [1,4].

As determined by the previous literature [42], the variables specific to psychotherapy refer to the idiosyncratic characteristics of each therapy modality, consisting mainly of the therapeutic approach and the techniques and procedures used. These items explain 11.17% of the variance.

The importance of these common factors in terms of their contribution to the effectiveness of therapy should be underlined [109]. Research even attributes 85% of the change in all psychotherapy modalities to these factors [24]. In fact, these three types of factors explain 51.53% of the variance in our scale.

Among all the common variables, it has been shown in the literature that interaction and therapeutic alliance are among those that contribute most to the effectiveness of psychological treatments [28,38]. Therefore, this dimension was initially included with only one item, “establishment of therapeutic alliance”, but the internal structure of the questionnaire through exploratory and confirmatory analysis unifies this item in a first factor that also includes “patient’s expectation of cure”, “patient’s faith and credibility assigned to the therapist”, “therapist’s perception of the patient’s involvement” and “therapist’s ability to influence the patient”. Therefore, a factor is found which is composed of two items referring to common therapist variables, two items referring to common patient variables and an item referring to the therapist–patient relationship. Considering the theoretical framework in which the variables are inserted, this factor would group the enhancers of the therapeutic alliance, that is, common variables of a perceptual order that favour such an alliance, and which are mainly related to the expectations or perceptions that both the therapist has of the patient and vice versa, and the interaction between the two.

On the other hand, it is confirmed that the common variables specific to the therapist are formed as one factor, including the “empathy shown by the therapist”, the “directivity and support shown by the therapist” and the “degree of acceptance, interest, understanding and encouragement shown by the therapist to the patient”, with strong emotional content. Similarly, the two items referring to the variables specific to psychotherapy, “therapeutic approach used” and “techniques or procedures used”, are included in a single factor.

Finally, one factor includes “therapist experience” and “patient’s involvement in the therapy”, with the first item corresponding to a common therapist variable and the second to a common patient variable. Thus, this factor would include two important common variables facilitating patient adherence to therapy and pointing to significant agents of change in the therapeutic process. Although the therapist’s experience has been a variable that has generated controversy in the literature, in the sense of whether it is an agent of change that acts directly or through the improvement it produces in other factors [78], the patient’s involvement in the treatments has been shown to be a direct agent of success in the intervention [50,52].

Exploratory and confirmatory analyses identified therapy-specific variables and common patient, therapist and interaction variables but divided into four different factors as follows: (1) therapeutic alliance enhancers; (2) psychotherapist emotional characteristics; (3) therapy-specific variables, and (4) facilitators of patient engagement with therapy. Consequently, the resulting 12-item scale, the Psychotherapeutic Effectiveness Attribution Questionnaire (PEAQ-12), is considered to be an enriching contribution to the study of psychotherapists for research and the development of training programmes.

In summary, it would be interesting to further investigate the validity of this questionnaire, its internal structure and its dimensions, using larger samples and in populations with varied psychotherapeutic orientations. It should be emphasised that the results should be interpreted with caution as the statistical power of confirmatory analysis with such a small sample may be limited. However, the results of this analysis have been included because they may be of value considering the pilot nature of the study. In addition, it would be a great contribution to associate, through this questionnaire, the attributions that therapists establish and the percentage of effect of the treatment on their patients. In fact, in this study, the interpretation of statistical results has been limited to what the therapists consider effective elements of psychotherapy and not to the factors that make the treatment effective, as the previous literature has already very extensively addressed the real effect of the different psychotherapeutic variables.

## Figures and Tables

**Table 1:** Fit indices suggested for the CFA.

Chi-Square Significance	Comparative Fit Index (CFI)	Tucker–Lewis Index (TLI or NNFI)	Root Mean Square Error of Approximation (RMSEA)	Standardised Root Mean Square Residual (SRMR)
If chi-square was not significant, the model had reached a perfect fit with the observed data.	Values ≥ 0.90 indicated a good fit [94].	Values ≥ 0.90 indicated a good fit [95].	Values ≤ 0.05 or 0.08 indicated a good fit, such as the upper bound of 90% confidence interval [96].	Values ≤ 0.05 indicated a good fit [97].

**Table 2:** Expert judgement. Congruence indices based on the objective congruence method (12 experts).

	Ijk
The therapeutic approach used	0.75
The techniques or procedures used	0.83
The patient’s expectations of cure	0.916
The patient’s involvement in the therapy	0.916
The patient’s faith and credibility assigned to the therapist	0.66
Emotional well-being of the therapist	0.16 (removed)
The empathy shown by the therapist	0.66
The directivity and support shown by the therapist	0.75
The therapist’s perception of the patient’s involvement	0.58
The therapist’s ability to influence the patient	0.83
The degree of acceptance, interest, understanding and encouragement shown by the therapist to the patient	0.83
The experience of the therapist	1
The establishment of a therapeutic alliance between the therapist and patient	0.66

**Table 3:** Position of items with score means and standard deviations and reliability results.

	Mean	SD	Cronbach’ Alpha if Item Deleted	Floor Effect “1 Point” (%)	Ceiling Effect “5 Points” (%)
1 Therapeutic approach used	3.79	1.003	0.723	2.9	27.5
2 Techniques or procedures used	4.11	0.935	0.729	1.5	36.8
3 Patient’s expectations of cure	3.98	0.907	0.690	1.6	36.8
4 Patient’s involvement in the therapy	4.59	0.663	0.708	0.0	66.7
5 Patient’s faith and credibility assigned to the therapist	3.71	0.906	0.698	0.0	21.7
6 Empathy shown by the therapist	4.16	0.807	0.696	1.9	40.6
7 Directivity and support shown by the therapist	3.59	1.026	0.701	3.0	19.4
8 Therapist’s perception of the patient’s involvement	3.41	1.026	0.676	4.4	16.2
9 Therapist’s ability to influence the patient	4.16	0.902	0.688	1.5	41.8
10 Degree of acceptance, interest, understanding and encouragement shown by the therapist to the patient	3.98	0.942	0.720	1.4	36.2
11 Experience of the therapist	4.25	0.861	0.740	0.0	50
12 Establishment of a therapeutic alliance between the therapist and patient	4.35	0.806	0.731	1	56.5

**Table 4:** Rotated component matrix of PCA.

	Component			
	1	2	3	4
1 The patient’s faith and credibility assigned to the therapist	0.755			
2 The therapist’s ability to influence the patient	0.736			
3 The patient’s expectations of cure	0.638			
4 The therapist’s perception of the patient’s involvement	0.614			
5 The establishment of a therapeutic alliance between the therapist and patient	0.529			
6 The empathy shown by the therapist		0.777		
7 The directivity and support shown by the therapist		0.762		
8 The degree of acceptance, interest, understanding and encouragement shown by the therapist to the patient		0.745		
9 The techniques or procedures used			0.911	
10 The therapeutic approach used			0.868	
11 The experience of the therapist				0.792
12 The patient’s involvement in the therapy				0.728

Extraction method: principal component analysis. Rotation method: varimax with Kaiser normalisation. a. Rotation converged in 5 iterations.

## Data Availability

The data presented in this study are available on request from the corresponding author. The data are not publicly available due to privacy issues.

## Appendix A

You will find some statements below. Read each statement carefully and think how those statements influence the improvement of a patient undergoing psychotherapy.

Please, check each item according to the following: 1 is “no influence at all” and 5 is “great influence”).

**Table A1:** Psychotherapeutic Effectiveness Attribution Questionnaire (PEAQ-12).

1 Therapeutic approach used (the theoretical orientation on which the psychotherapy is based)	1	2	3	4	5
2 Techniques or procedures used	1	2	3	4	5
3 Patient’s expectations of cure	1	2	3	4	5
4 Patient’s involvement in the therapy	1	2	3	4	5
5 Patient’s faith and credibility in the therapist	1	2	3	4	5
6 Empathy shown by the therapist	1	2	3	4	5
7 Directivity and support shown by the therapist	1	2	3	4	5
8 Therapist’s perception of the patient’s involvement	1	2	3	4	5
9 Therapist’s ability to influence the patient	1	2	3	4	5
10 Degree of acceptance, interest, understanding and encouragement shown by the therapist to the patient	1	2	3	4	5
11 Experience of the therapist	1	2	3	4	5
12 Establishment of a therapeutic alliance between the therapist and patient	1	2	3	4	5
